# Modulation of potassium channels by transmembrane auxiliary subunits via voltage‐sensing domains

**DOI:** 10.14814/phy2.15980

**Published:** 2024-03-19

**Authors:** Koichi Nakajo, Go Kasuya

**Affiliations:** ^1^ Division of Integrative Physiology, Department of Physiology Jichi Medical University Shimotsuke Japan

**Keywords:** auxiliary subunits, cryo‐EM, KCNQ1, K_V_, potassium channels, Slo1

## Abstract

Voltage‐gated K^+^ (K_V_) and Ca^2+^‐activated K^+^ (K_Ca_) channels are essential proteins for membrane repolarization in excitable cells. They also play important physiological roles in non‐excitable cells. Their diverse physiological functions are in part the result of their auxiliary subunits. Auxiliary subunits can alter the expression level, voltage dependence, activation/deactivation kinetics, and inactivation properties of the bound channel. K_V_ and K_Ca_ channels are activated by membrane depolarization through the voltage‐sensing domain (VSD), so modulation of K_V_ and K_Ca_ channels through the VSD is reasonable. Recent cryo‐EM structures of the K_V_ or K_Ca_ channel complex with auxiliary subunits are shedding light on how these subunits bind to and modulate the VSD. In this review, we will discuss four examples of auxiliary subunits that bind directly to the VSD of K_V_ or K_Ca_ channels: KCNQ1–KCNE3, Kv4‐DPP6, Slo1‐β4, and Slo1‐γ1. Interestingly, their binding sites are all different. We also present some examples of how functionally critical binding sites can be determined by introducing mutations. These structure‐guided approaches would be effective in understanding how VSD‐bound auxiliary subunits modulate ion channels.

## 1 INTRODUCTION

Voltage‐gated K^+^ (K_V_) channels play many physiological roles in the human body. The K_V_ channel α subunit is a six‐transmembrane protein, and four α subunits form a single K_V_ channel. They are activated by membrane depolarization. The membrane potential is sensed by the fourth transmembrane segment (S4) of the voltage‐sensing domain (VSD), which consists of the first four transmembrane segments (S1–S4). Because S4 has several positively charged amino acid residues (mostly arginine) every three amino acid residues, membrane depolarization induces upward movement of S4, resulting in the opening of the pore domain (PD; S5–S6). Action potential repolarization is the best‐known function of K_V_ channels. They even play physiological roles in K^+^ transport or K^+^ homeostasis in non‐excitable cells such as epithelial cells. K_V_ channels sometimes become voltage‐insensitive when associated with auxiliary subunits in these cells. Ca^2+^‐activated K^+^ (K_Ca_) channels are similar to K_V_ channels as a molecule, but they are activated not only by depolarization but also by intracellular Ca^2+^ binding. They share a common structure with VSD and PD and are recognized as close family members of Kv channels.

In the human genome, at least 40 K_V_ channel genes from 12 subfamilies from K_V_1 to K_V_12, and eight genes for K_Ca_ channels have been identified. This gene diversity far exceeds the diversity of voltage‐gated Na^+^ (Na _V_ ) and voltage‐gated Ca^2+^ (Ca_V_) channels, which contain 9 and 10 genes, respectively. One of the reasons for the diversity of K_V_ and K_Ca_ genes may be to confer a wide variety of firing properties to neurons and muscles. In addition, there are several auxiliary subunits for K_V_ and K_Ca_ channels. Among them, a few auxiliary subunits bind directly to the VSD. Since K_V_ and K_Ca_ channels are regulated by membrane potential through VSDs, it is reasonable to modulate the channels by directly binding to the VSD.

**FIGURE 1:**
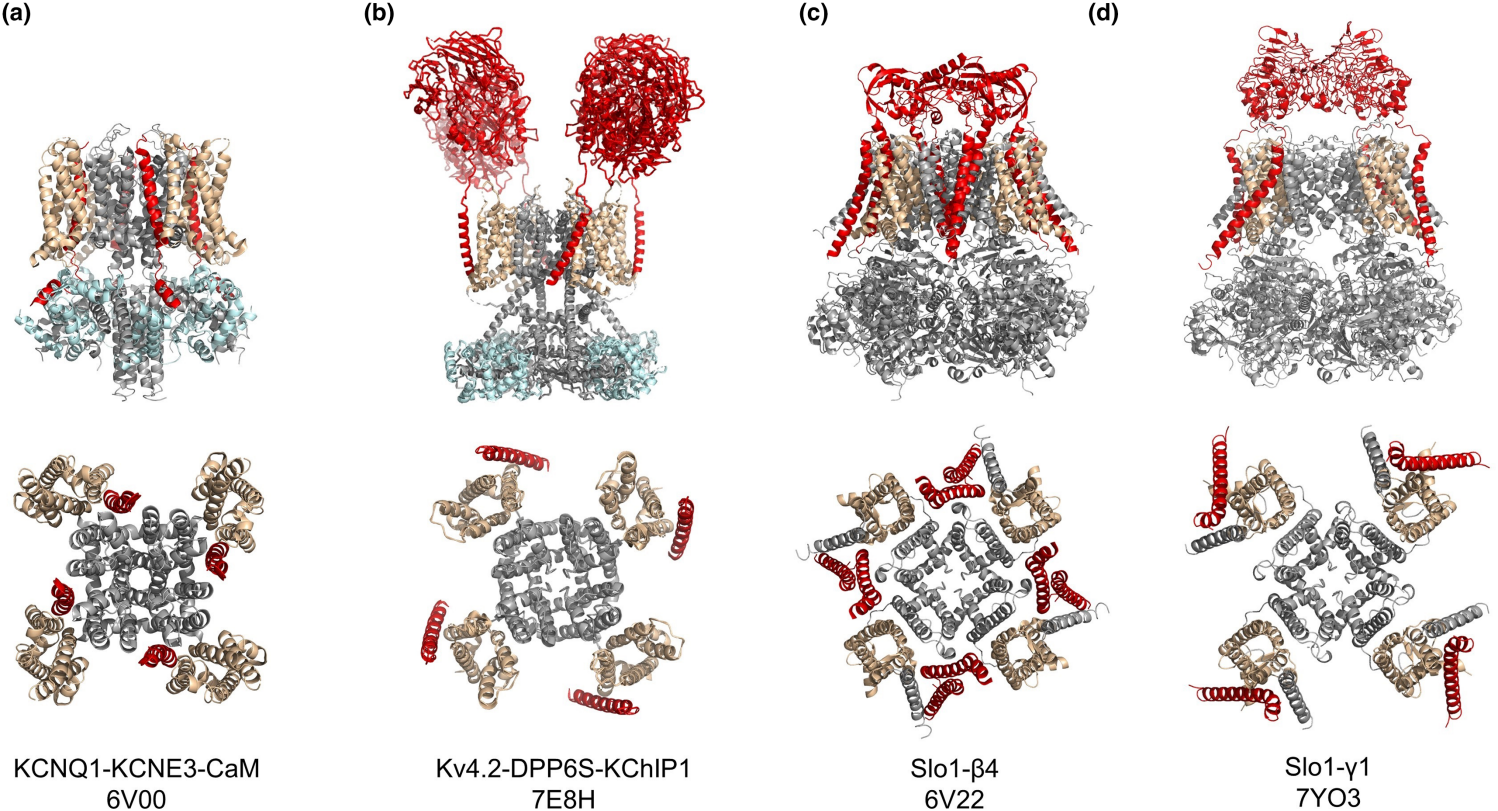
Lateral and top views of voltage‐gated potassium channel complexes with transmembrane auxiliary subunits. (a) KCNQ1‐KCNE3‐CaM (pdb:6V00), (b) Kv4.2‐DPP6S‐KChIP1 (pdb:7E8H), (c) Slo1‐β4 (pdb:6V22), and (d) Slo1‐γ1 (LRRC26) (pdb:7YO3). Transmembrane auxiliary subunits are colored in red. Voltage‐sensing domains (S1–S4) are colored in gold. Cytoplasmic auxiliary subunits (CaM and KChIP1) are colored in blue. In the top views, the extracellular and intracellular domains are omitted to emphasize the transmembrane structures.

Three ion channels are presented in this review: KCNQ1 (Kv7.1), Kv4, and Slo1 (BK) channels, all of which are modulated by transmembrane auxiliary subunits that bind directly to the VSD (Figure 1). Using recent cryo‐EM structures of these ion channel complexes, we will discuss how their auxiliary subunits modulate the gating properties of the channels.

## 2 KCNQ1 (Kv7.1) CHANNEL AND KCNE SUBUNITS

The KCNQ1 channel is a voltage‐gated potassium (K_V_) channel and was the first member of the KCNQ channel family to be discovered. While the other KCNQ channel genes (*KCNQ2*–*KCNQ5*) are expressed primarily in excitable cells, such as neurons and smooth muscle cells, the *KCNQ1* gene is widely expressed in the human body, including the heart, kidney, intestine, and inner ear. *KCNQ1* (then named *KVLQT1*) gene was first reported as a causative gene of long QT syndrome (Wang et al., 1996). Soon after, KCNE1 (minK) was identified as an auxiliary subunit for the KCNQ1 channel (Barhanin et al., 1996; Sanguinetti et al., 1996). KCNE1 (minK) was initially cloned from rat kidney mRNA as a single transmembrane protein of 129 amino acids long (Takumi et al., 1988). When the RNA of KCNE1 was injected into *Xenopus* oocytes, it induced a slowly activating K^+^ current in oocytes. It later turned out that the exogenous KCNE1 gene could upregulate the endogenous (*Xenopus*) KCNQ1 channels.

**FIGURE 2:**
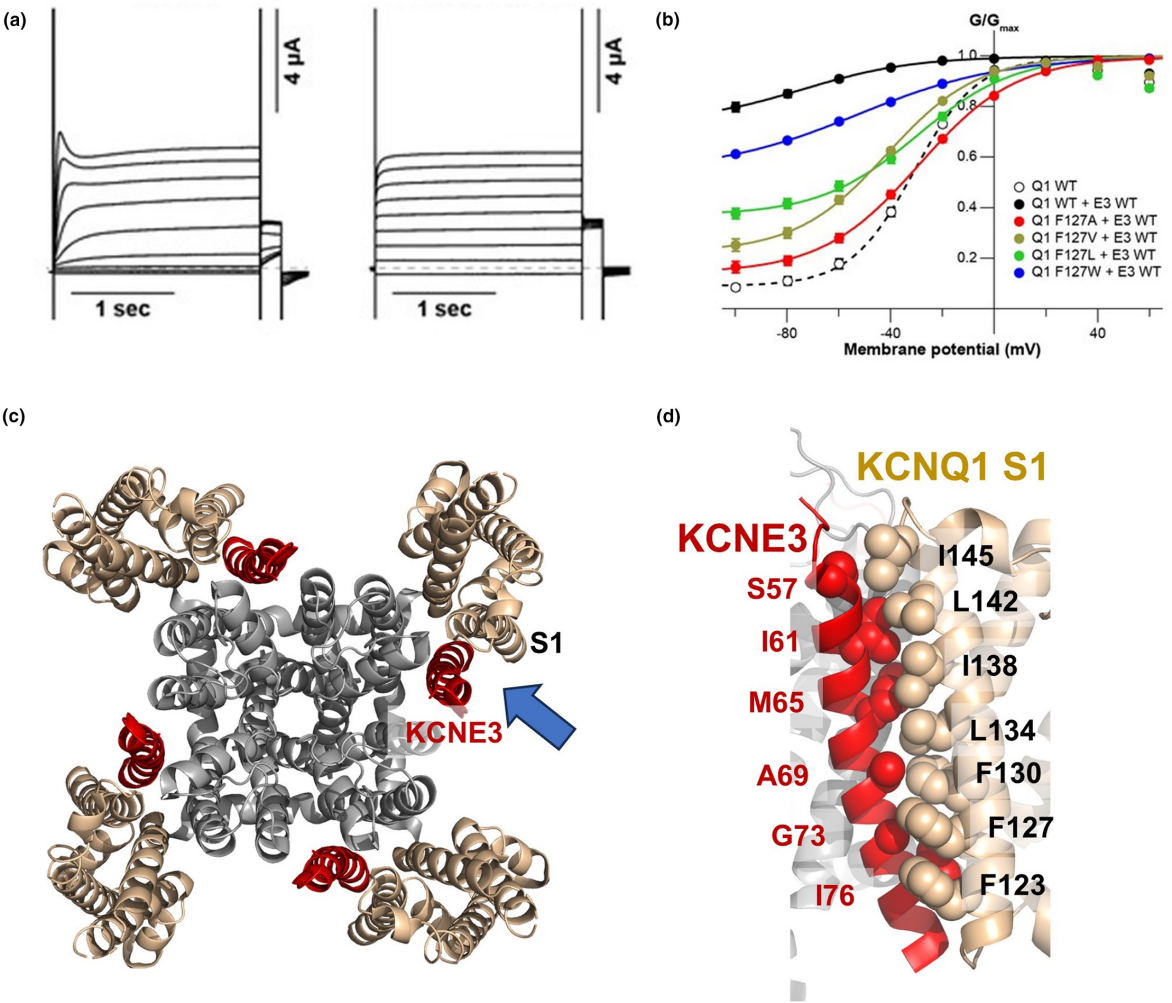
(a) Representative current traces for KCNQ1 alone (left) and KCNQ1 + KCNE3 (right). (b) G–V relationships for KCNQ1, KCNQ1 + KCNE3, and KCNQ1 + KCNE3 F127 mutants. (a) and (b) are reproduced from Kasuya and Nakajo (2022). (c) Top view of the KCNQ1‐KCNE3 complex. KCNE3 is shown in red. The VSDs are shown in gold. The blue arrow indicates the angle of (d). (d) Interaction surface of KCNQ1 S1 and KCNE3. Interacting amino acid residues are highlighted as spheres.

KCNQ1 channel can produce voltage‐dependent outward K^+^ current as a homotetramer. However, the co‐expression of KCNE1 causes many changes in KCNQ1 currents, including larger current amplitude, slower activation/deactivation kinetics, loss of inactivation, positively shifted voltage dependence, ion selectivity, and pharmacology. As the current of the KCNQ1–KCNE1 co‐expression resembled the slow delayed rectifier K^+^ current (I_Ks_) in the human ventricle, it was believed that KCNQ1 and KCNE1 were co‐expressed in human ventricular myocytes (Jost et al., 2007). After recognizing KCNE1 as an auxiliary subunit of KCNQ1, four other KCNE‐related genes (*KCNE2‐5*) were identified in the human genome (Abbott et al., 1999, 2001; Abbott & Goldstein, 1998; Angelo et al., 2002; Grunnet et al., 2002; Schroeder et al., 2000). Although KCNE2 and KCNE3 were first identified as auxiliary subunits for other K_V_ channels (hERG (Kv11.1) and Kv3.4), all KCNE genes are expressed in the human heart and can modulate KCNQ1 channels (Lundquist et al., 2005). As described above, KCNE1 makes the KCNQ1 channel the slowly activating I_Ks_ channel, while KCNE3 makes it a constitutively open channel (Figure 2a,b). KCNE2 also makes the KCNQ1 channel constitutively active but with a lower current. KCNE4 completely inhibits the KCNQ1 current, and KCNE5 also inhibits it by shifting the G–V curve in the far positive direction. Because of the drastic and diversified gating modulation for the KCNQ1 channel, the modulation mechanisms by KCNE1 and KCNE3 have been intensively studied. When KCNE1 binds to KCNQ1, the kinetics of KCNQ1 becomes significantly slower (approximately 100 times slower in activation and deactivation kinetics), and the voltage dependence (G–V relationship) is shifted to the far positive direction (about 50 mV), indicating that the KCNQ1 channel becomes more difficult to open. On the other hand, KCNE3 results in a KCNQ1 channel that remains constitutively open. Thus, the gating properties of the two KCNE proteins appear to be opposite from each other.

Melman and colleagues proposed that the group of three amino acid residues (“FTL” in KCNE1 and “TVG” in KCNE3) located in the middle of the transmembrane segment determine the opposing modulation of KCNE proteins (Melman et al., 2001, 2002). Later, it was shown that KCNE1 binds directly to the pore domain and that the triplet and some of the amino acid residues on S6 may interact (Melman et al., 2004; Panaghie et al., 2006).

Although KCNE1 binds to the pore domain, it could simultaneously bind to and affect the VSD. To track the voltage sensor movement in the presence of KCNE proteins, MTS reagents were used, which can covalently modify cysteine side chains and alter the channel properties (Larsson et al., 1996). Two groups introduced a cysteine residue at the top of the S4 segment (e.g., A226C) and showed that KCNE1 and KCNE3 alter the reaction rate of MTS reagents, suggesting that KCNE proteins change VSD movement (Nakajo & Kubo, 2007; Rocheleau & Kobertz, 2008). Then, the VSD movement of the KCNQ1 channel was directly examined by voltage‐clamp fluorometry (VCF) (Osteen et al., 2010). The first VCF report confirmed that KCNE1 altered VSD movements and showed that the fluorescence–voltage (F–V) relationship was split into two components, faster F_1_ and slower F_2_. Later, it was found that KCNE1 stabilizes the intermediate state between the down and up states of the S4 segment (Barro‐Soria et al., 2014). Because KCNE1 eliminates the intermediate open (IO) state, which is an ion‐permeable state with the VSD at the intermediate position for the KCNQ1 channel, the G–V curve of the KCNQ1–KCNE1 channel follows the F_2_ component of the F–V relationship (Hou et al., 2017; Zaydman et al., 2014).

In 2017, the first cryo‐EM structure of the KCNQ1 channel with calmodulin has been reported (Sun & MacKinnon, 2017). The VSDs are considered to be in the up state with no negative membrane potential, but the pore domain is closed because it does not contain PIP_2_, which is required for channel opening (Zaydman et al., 2013; Zhang et al., 2003). Three years later, the first cryo‐EM structure of the KCNQ1 channel complex with KCNE proteins (KCNE3) has been published (Sun & MacKinnon, 2020). Each KCNE3 protein binds to the VSD and the pore domain in this structure, so four KCNE3 proteins are included in the complex (Figure 2c). That is consistent with the stoichiometry of the KCNQ1–KCNE1 complex, which can have up to four KCNE1 subunits in the complex at least in expression systems (Murray et al., 2016; Nakajo et al., 2010). The structure with PIP_2_ (PDB: 6V01) is assumed to be in the activated open (AO) state, not the IO state, because the fourth arginine R4 (R237) of S4 is located above the hydrophobic plug (also serves as a charge transfer center) (Taylor et al., 2020). In this structure, the triplet (“TVG”) faces three different helices: T71 faces S5 (I263), V72 faces S1 (F130) and S4 (V241), and G73 faces S1 (F127). The lower part of KCNE3 (L75 and Y79) interacts with the lower part of S4 (the main chain of R243), so this interaction may be necessary to keep the S4 segment in the up state.

While KCNE3 partially interacts with S4, it interacts with the entire transmembrane domain of the S1 segment (Figure 2c,d) (Sun & MacKinnon, 2020). We, therefore, investigated whether this interaction is functionally significant for the constitutive openness induced by the KCNE3 interaction (Kasuya & Nakajo, 2022). We mutated every single amino acid residue in the interaction face between KCNQ1 S1 and KCNE3 to different sizes of hydrophobic amino acid residues. Figure 2b shows an example of G–V relationships from F127 mutants. Interestingly, for almost every amino acid residue we tested, the constitutive activity depended on how different the size of the introduced amino acid side chain was from the wild type: the larger the difference, the weaker the constitutive activity (smaller currents at −100 mV in Figure 2b). Subsequent VCF experiments showed that these mutations destabilized the intermediate state, indicating that the tight binding of KCNE3 to the S1 segment is required to stabilize the intermediate (open) state. This may be at least one of the mechanisms by which the KCNQ1–KCNE3 channel remains open in the physiological range of membrane potential.

So, what about KCNE1? What part of KCNQ1 does KCNE1 bind? Although the cryo‐EM structure of the KCNQ1–KCNE1 complex is not available yet, KCNE1 may similarly bind to the KCNQ1 channel as KCNE3 does (Kang et al., 2008; Nakajo et al., 2011; Nakajo & Kubo, 2007). Our preliminary data suggest that KCNE1 may also bind to the S1 segment of KCNQ1 (Kasuya & Nakajo, 2022). More detailed information is needed to elucidate the modulation mechanism of KCNE1, especially because the KCNQ1–KCNE1 channel should have at least three states at physiological membrane potential: closed state, intermediate (closed) state, and open state. Therefore, the binding mode of KCNE1 may differ depending on the states (Kang et al., 2008).

## 3 Kv4 CHANNEL AND DPP SUBUNITS

Kv4 channel is a family member of K_V_ channels. So, its molecular architecture is similar to other *Shaker*‐type potassium channels, Kv1‐3. The Kv4 (*Shal*) gene was initially isolated from the *Drosophila* cDNA library by using a cDNA probe of the first Kv channel *Shaker* along with Kv2 (*Shab*) and Kv3 (*Shaw*) (Butler et al., 1989; Wei et al., 1990). One of the characteristics of the Kv4 current is its rapid inactivation. It was first described as a transient outward current, “A‐type current” or I_A_, in molluscan neurons (Connor & Stevens, 1971; Hagiwara et al., 1961; Nakajima, 1966). I_A_ significantly impacts the excitability of neurons by activating and inactivating in the subthreshold range of the neuron. Therefore, the expression and kinetic properties of Kv4 may substantially influence the neuron's excitability. In the heart, the expression of Kv4.3 and its auxiliary subunit Kv channel‐interacting protein 2 (KChIP2) is higher in epicardial cells than in endocardial cells. Because KChIP2 increases current amplitude and slows inactivation of Kv4.3 current, the epicardial cell has a shorter action potential duration. This mechanism contributes to the transmural difference in cardiac excitability (Fedida & Giles, 1991; Wang et al., 2022; Zicha et al., 2004).

**FIGURE 3:**
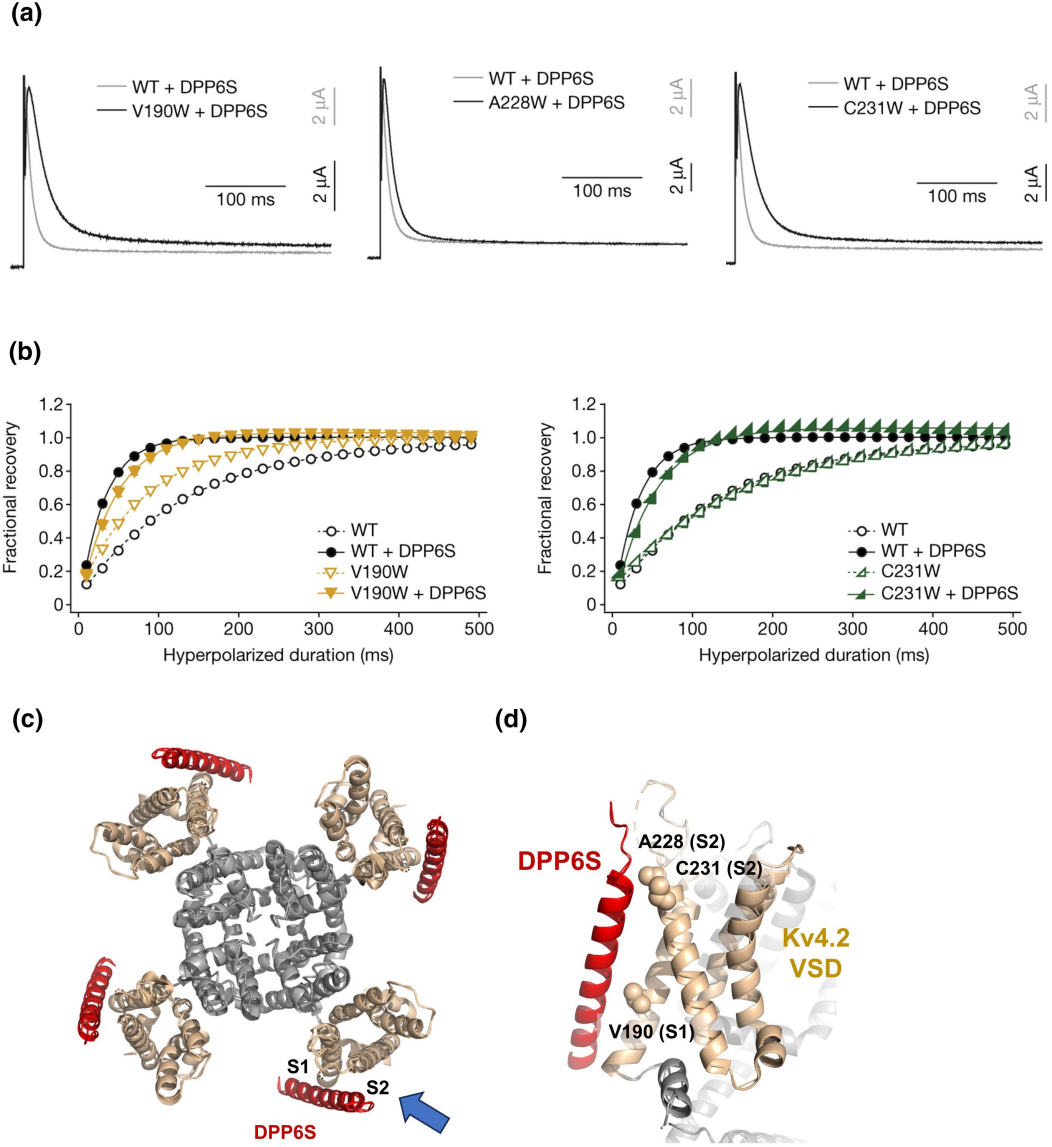
(a) Representative current traces for Kv4 WT + DPP6S and Kv4 mutants (V190W, A228W, and C231W) + DPP6S. (b) Recovery from inactivation of Kv4 WT and Kv4 mutants (V190W and C231W) in the presence and absence of DPP6S. (a) and (b) are reproduced from Kise et al. (2021). (c) Top view of the Kv4‐DPP6S complex. DPP6S is shown in red. The VSDs are shown in gold. The blue arrow indicates the angle of (d). (d) Interaction surface of Kv4.2 VSD and DPP6S. Functionally interacting amino acid residues are highlighted as spheres.

Two major auxiliary subunits for the Kv4 channel are known. One is the abovementioned KChIP family (KChIP1‐4), and the other is dipeptidyl‐peptidase 6/10 (DPP6/10). KChIP subunits increase current amplitude, slow inactivation kinetics, and facilitate recovery from inactivation (An et al., 2000). KChIPs are cytoplasmic proteins, and up to four KChIPs bind to the cytoplasmic domain of Kv4 to modulate the channel (Kitazawa et al., 2014; Pioletti et al., 2006; Wang et al., 2007). DPP6 and DPP10 are members of the dipeptidyl‐peptidase IV family and are single transmembrane proteins with a large extracellular C‐terminal pseudo‐peptidase domain. They bind to the transmembrane region of Kv4 channels, increasing current amplitude, accelerating activation/inactivation kinetics and recovery from inactivation (Foeger et al., 2012; Jerng et al., 2004; Nadal et al., 2003; Zagha et al., 2005) (Figure 3a,b). DPP6 (and DPP10) form a homodimer and can be expressed by itself (Bezerra et al., 2015; Foeger et al., 2012; Kitazawa et al., 2015; Strop et al., 2004). Probably because they exist as a dimer, the stoichiometry of Kv4‐DPP tends to be 4:2 or 4:4 (Kise et al., 2021; Kitazawa et al., 2015).

In 2021 and 2022, three groups independently reported cryo‐EM structures of Kv4 with the auxiliary subunits (Kise et al., 2021; Ma et al., 2022; Ye et al., 2022). The transmembrane region of DPP6 directly binds to two segments, S1 and S2: The lower part of DPP6 interacts with the S1, and the upper part of DPP6 interacts with the S2 (Figure 3c,d). As the deletion of the extracellular C‐terminal domain of DPP6 does not affect the modulation, the transmembrane region is essential for channel modulation (Kitazawa et al., 2015; Lin et al., 2014; Ye et al., 2022).

We mutated some of the interacting amino acid residues on the S1 and S2 segments. Since alanine substitutions were not sufficient to affect DPP6 modulation, we introduced a bulky tryptophan residue one at a time to evaluate functional interactions. We identified three tryptophan mutants (V190W in S1; A228W and C231W in S2) that showed slow inactivation in the presence of DPP6, suggesting that they inhibit DPP6 function by preventing interaction (Figure 3a,d) (Kise et al., 2021). V190W and C231W also showed slower recovery from inactivation in the presence of DPP6 (Figure 3b,d). Ye et al. also reported that the rigidity of DPP6 may be important for the modulation. They made a double glycine substitution V41G/I42G and I42G/C43G on DPP6. These mutants showed impaired modulation of Kv4.2 currents (Ye et al., 2022). These structure‐based functional analyses support the idea that the interaction between DPP6/10 and S1/S2 segments is necessary for proper modulation by DPP6/10. However, these analyses did not answer why the interaction between the transmembrane segments can modulate the inactivation properties. Future analysis of the VSD motion using VCF or gating current recordings (Dougherty et al., 2008) may be required.

## 4 Slo1 CHANNEL AND β/γ SUBUNITS

Slo1 channels are large conductance, voltage‐ and Ca^2+^‐activated K^+^ channels. They exhibit characteristic large single‐channel conductance in the 100–300 pS range and are called BK (big potassium) or MaxiK (Adelman et al., 1992; Butler et al., 1993; Pallotta et al., 1981). Slo1 channels are expressed throughout the human body, including neurons, muscle cells, and non‐excitable cells, so they are involved in many physiological functions. For example, Slo1 channels facilitate hyperpolarization after action potentials and contribute to the fast after‐hyperpolarization (fAHP) (Shao et al., 1999). They are also activated by local Ca^2+^ release from ryanodine receptors in smooth muscle cells as spontaneous transient outward currents (STOCs) to relax smooth muscle cells (Bolton & Imaizumi, 1996).

The Slo1 channel belongs to the large family of K_V_ channels. Therefore, its molecular architecture is the similar to that of other K_V_ channels: The S1–S4 segments form the VSD, and the S5–S6 segments contain the pore domain, except that it has the S0 segment before the S1 segment. It should also be noted that Slo1 has a non‐domain‐swapped structure where the VSD and PD of the same subunit contact each other. This is quite different from the domain‐swapped structure of other Kv channels, including Kv7 and Kv4. Therefore, Slo1 may have a different voltage‐dependent gating machinery compared to the rest of the Kv channel family. The Slo1 channel has two intracellular regulators of K^+^ conductance (RCK1 and RCK2), where Ca^2+^ directly binds (Yang et al., 2015). Mg^2+^ binds to the VSD and RCK1 interface, which connects the voltage sensor and the Ca^2+^ sensor (Shi et al., 2002; Shi & Cui, 2001).

The Slo1 channel has two major auxiliary subunits, which can bind to the VSD. One is the β subunit (β1–β4), which has two transmembrane regions (Orio et al., 2002). The other major group is the γ subunit (γ1–γ4). The γ subunits have a single transmembrane region and a large extracellular leucine‐rich repeat (LRR) domain. Thus, they were initially identified from LRR‐containing (LRRC) proteins as γ1 (LRRC26), γ2 (LRRC52), γ3 (LRRC55), and γ4 (LRRC38) (Yan & Aldrich, 2010, 2012). Newly identified Slo1 channel regulatory proteins of the LINGO family (LINGO1‐4) also have a single transmembrane region and contain the LRR domain (Dudem et al., 2020, 2023). They are all known to modulate many gating properties, including voltage dependence of activation, activation/deactivation kinetics, Ca^2+^ sensitivity, inactivation, and pharmacological properties.

**FIGURE 4:**
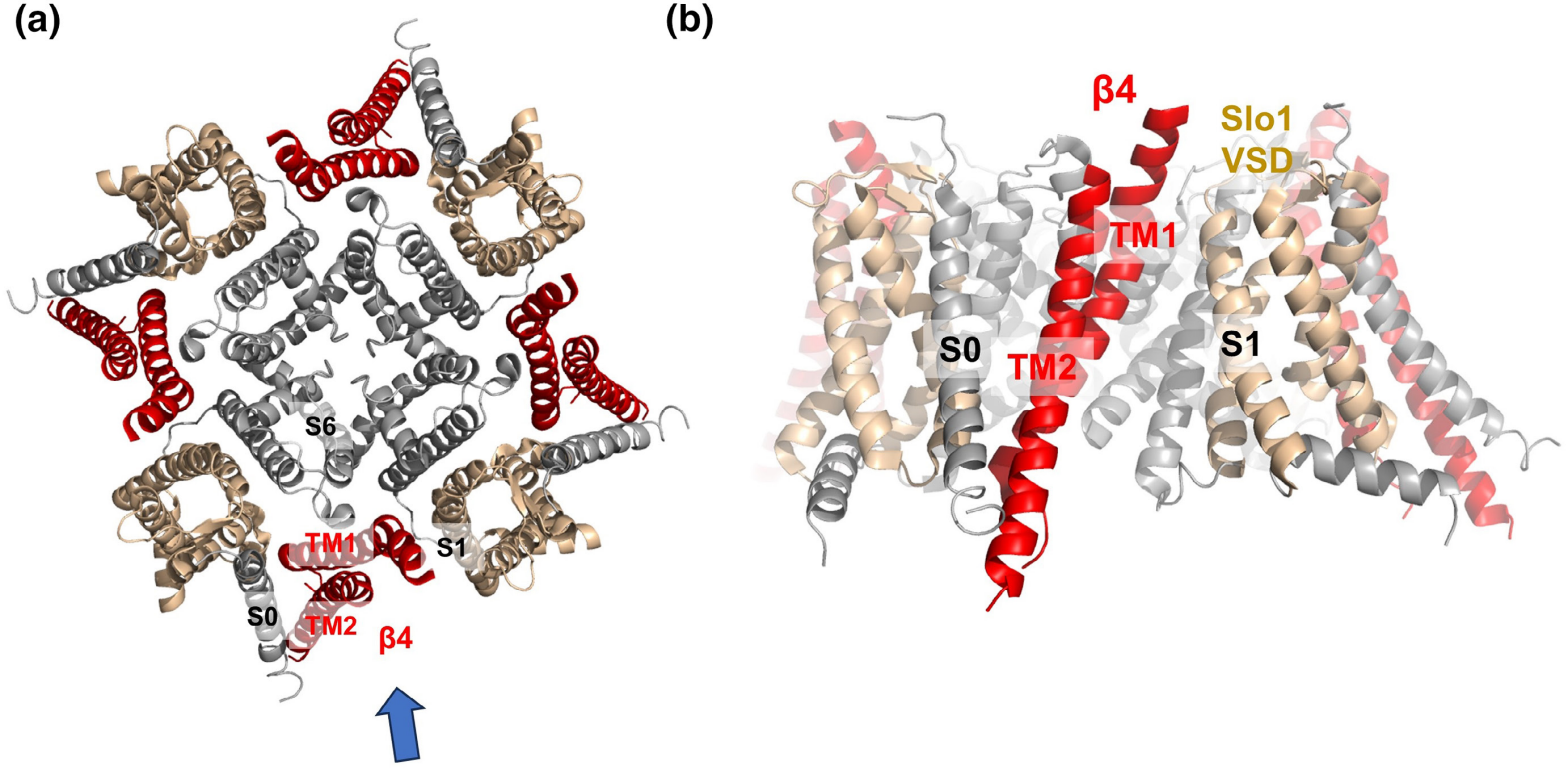
(a) Top view of the Slo1‐β4 complex. β4 is shown in red. The VSDs, but not S0, are shown in gold. The blue arrow indicates the angle of (b). (b) Side view of the Slo1‐β4 complex.

The first complete Slo1 channel cryo‐EM structure was reported in 2017 (Hite et al., 2017; Tao et al., 2017). Soon after, the first Slo1 channel complex containing β4 subunits was reported (Figure 1c) (Tao & MacKinnon, 2019). The β4 subunit has two transmembrane segments (TM1 and TM2). In the cryo‐EM structure, TM1 binds directly to two segments, S1 and S6 (Figure 4). The bottom of TM1 (intracellular side) contacts the S2–S3 linker, S0, and the S6‐RCK1 linker. TM2 is lined next to the S0 segment. Tao and MacKinnon made a series of chimeras between β1 and β4 and identified the N‐terminus of TM1 and the cytoplasmic N‐terminal region as critical for modulating the activation/deactivation kinetics of Slo1 channels (Tao & MacKinnon, 2019). The β4 subunit also has a large extracellular (EC) domain. Although it does not directly interact with the α subunit of the Slo1 channel according to the structure, the previous mutant experiments suggest that the EC domain may also contribute to gating modulation (Gruslova et al., 2012).

**FIGURE 5:**
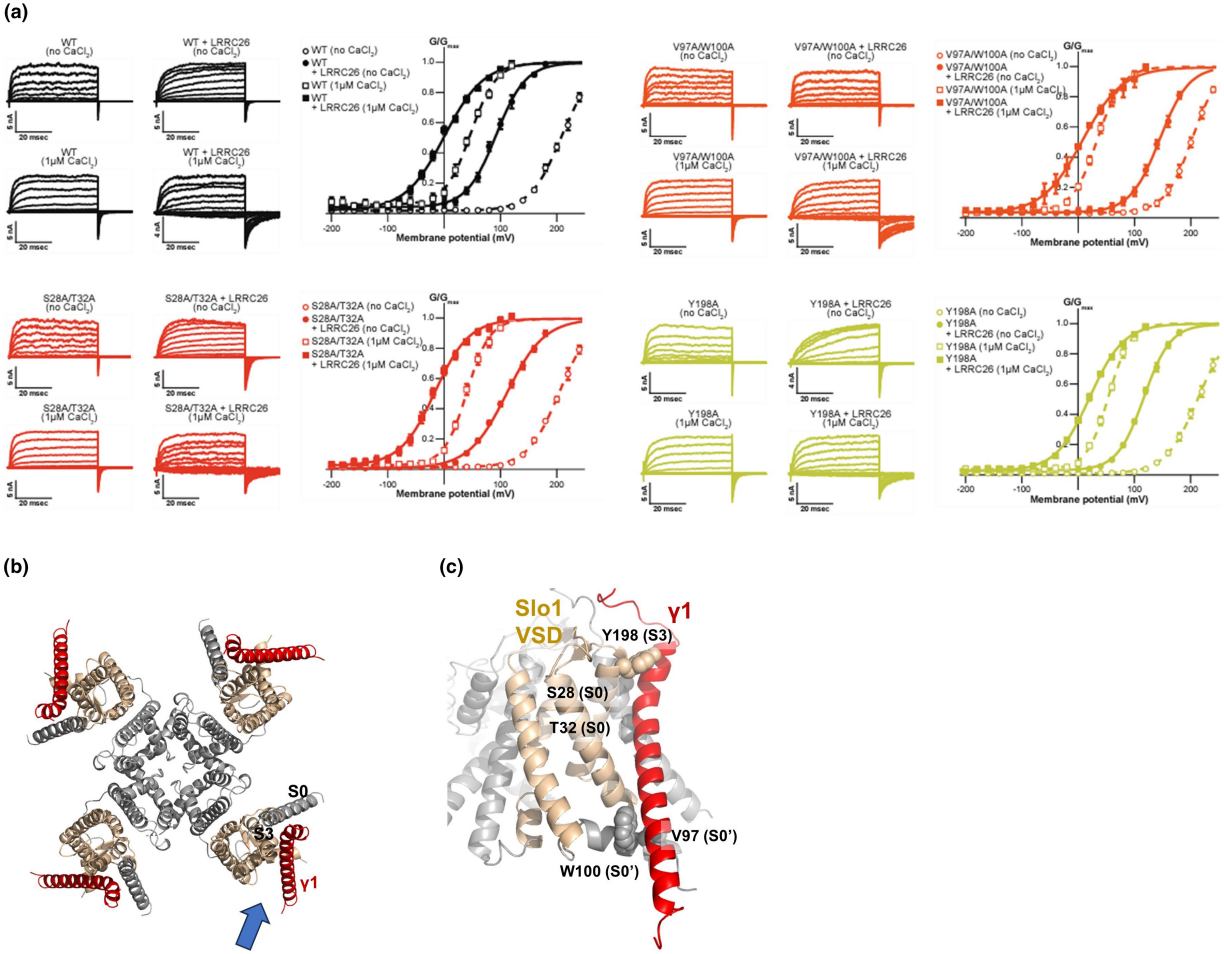
(a) Representative current traces for Slo1 alone and Slo1‐γ1 (LRRC26) in 0 and 1 μM intracellular Ca^2+^ (left) and the G–V relationships (right). Slo1 WT (black), Slo1 S28A/T32A double mutant (red), Slo1 V97A/W100A double mutant (orange), and Slo1 Y198A mutant (yellow) are shown. (a) is reproduced from Yamanouchi et al. (2023). (b) Top view of the Slo1‐γ1 (LRRC26) complex. γ1 (LRRC26) is shown in red. The VSDs, but not S0, are shown in gold. The blue arrow indicates the angle of (c). (c) Interaction surface of Slo1 VSD and γ1 (LRRC26). Functionally interacting amino acid residues are highlighted as spheres.

Among the modulation by γ subunits, γ1 (LRRC26) has the most prominent effect on the activation curves of Slo1 channels. While each γ subunit negatively shifts the G–V curves, γ1 subunits shift the G–V by an ~−140 mV; thus, activation of the Slo1‐γ1 (LRRC26) channel may not require Ca^2+^ or depolarization (Figure 5a) (Yan & Aldrich, 2010, 2012). The cryo‐EM structure of the Slo1‐γ1 (LRRC26) channel complex was recently determined (Yamanouchi et al., 2023), and two other papers on similar structures have been submitted to bioRxiv (Kallure et al., 2023; Redhardt et al., 2023). In this structure, the γ1 subunit interacts extensively with the VSD domain, including S0, the S0–S1 linker, S2, and S3 (Figure 5b,c). We mutated some interacting amino acid residues on the Slo1 channel side and confirmed that these interactions are required for the G–V shift induced by γ1 subunits (Figure 5a). The γ1 subunit also interacts with the RCK1 domain. These multiple interactions may allow the γ1 subunit to dually modulate the dependence on membrane potential and intracellular Ca^2+^.

As mentioned above, LRRC26 has a large extracellular LRR domain. Like the EC domain of the β4 subunit, the LRR domain does not directly contact Slo1. However, initial experiments with deletion mutants suggest that the LRR domain is necessary for proper gating modulation (Yan & Aldrich, 2010). Chimeric molecules of the γ1 subunit that replaced the LRR domain with a non‐LRR domain showed that the LRR domain itself does not have the ability to modulate gating, although it is necessary for the “all‐or‐none” modulation mechanism (Chen et al., 2022; Gonzalez‐Perez et al., 2014).

Notably, while all γ subunits negatively shift the G–V curves of the Slo1 channel, γ2‐4 subunits induce much smaller shifts than the γ1 subunit (Yan & Aldrich, 2012), suggesting that γ2‐4 subunits may have different modulation mechanisms for Slo1 channels. Future structural and biophysical analyses are needed to dissect the functional differences among γ subunits.

## 5 CONCLUSION

Kv channels are regulated by membrane potential through four VSDs. Therefore, it is a reasonable strategy for regulatory subunits to bind directly to the VSD. We have reviewed four examples of transmembrane auxiliary subunits that bind directly to the VSD of K_V_ or K_Ca_ channels. All of them basically bind to non‐S4 segments to regulate gating, probably because direct binding to the S4 segment may be too drastic to modulate the channels. With new ion channel structures being published monthly, we get a clearer idea of how auxiliary subunits modulate channel gating.

## AUTHOR CONTRIBUTIONS

KN and GK wrote the manuscript.

## FUNDING INFORMATION

This work was supported by JSPS KAKENHI (21K06786 to KN).

## ETHICS STATEMENT

Not applicable.
